# Consistency of linkage disequilibrium between Chinese and Nordic Holsteins and genomic prediction for Chinese Holsteins using a joint reference population

**DOI:** 10.1186/1297-9686-45-7

**Published:** 2013-03-21

**Authors:** Lei Zhou, Xiangdong Ding, Qin Zhang, Yachun Wang, Mogens S Lund, Guosheng Su

**Affiliations:** 1 Department of Molecular Biology and Genetics, Aarhus University, Tjele DK-8830, Denmark; 2 College of Animal Science and Technology, China Agricultural University, Beijing 100193, China

## Abstract

**Background:**

In China, the reference population of genotyped Holstein cattle is relatively small with to date, 80 bulls and 2091 cows genotyped with the Illumina 54 K chip. Including genotyped Holstein cattle from other countries in the reference population could improve the accuracy of genomic prediction of the Chinese Holstein population. This study investigated the consistency of linkage disequilibrium between adjacent markers between the Chinese and Nordic Holstein populations, and compared the reliability of genomic predictions based on the Chinese reference population only or the combined Chinese and Nordic reference populations.

**Methods:**

Genomic estimated breeding values of Chinese Holstein cattle were predicted using a single-trait GBLUP model based on the Chinese reference dataset, and using a two-trait GBLUP model based on a joint reference dataset that included both the Chinese and Nordic Holstein data.

**Results:**

The extent of linkage disequilibrium was similar in the Chinese and Nordic Holstein populations and the consistency of linkage disequilibrium between the two populations was very high, with a correlation of 0.97. Genomic prediction using the joint versus the Chinese reference dataset increased reliabilities of genomic predictions of Chinese Holstein bulls in the test data from 0.22, 0.15 and 0.11 to 0.51, 0.47 and 0.36 for milk yield, fat yield and protein yield, respectively. Using five-fold cross-validation, reliabilities of genomic predictions of Chinese cows increased from 0.15, 0.12 and 0.15 to 0.26, 0.17 and 0.20 for milk yield, fat yield and protein yield, respectively.

**Conclusions:**

The linkage disequilibrium between the two populations was very consistent and using the combined Nordic and Chinese reference dataset substantially increased reliabilities of genomic predictions for Chinese Holstein cattle.

## Background

Genomic selection was proposed in 2001 [1] and has since then become a major research topic in animal breeding. Accuracy of genomic prediction depends greatly on the size of the reference population [2,3]. The larger the reference population, the more accurate genomic prediction is. It was reported that reliabilities of genomic prediction of Holstein cattle increased when Holstein cattle of other countries were added to the reference dataset [4-6]. Similarly, pooling genotypes from other countries or populations to form a common reference population helped to increase the reliability of predictions in Brown Swiss cattle [6,7]. In addition, reliabilities of genomic prediction obtained by combining the reference populations of Danish, Swedish and Finnish red cattle were higher than those using single-country reference populations [8]. Holstein dairy cattle in China were originally imported from Europe and North America and were mostly derived from cross-breeding between the local yellow cattle and imported foreign Holstein bulls. It is assumed that the current Chinese Holstein population is genetically close to the other Holstein populations in the world. To date, the reference population of genotyped Holstein cattle in China is relatively small and includes mainly cows. It is expected that a joint reference dataset that combines Chinese Holstein cattle and Holstein cattle from other populations will greatly improve the reliability of genomic predictions of the Chinese Holstein population, assuming linkage disequilibrium between markers and quantitative trait loci (QTL) is consistent between the populations.

The objectives of this study were to: (1) estimate the consistency of linkage disequilibrium between the Chinese and the Nordic Holstein populations and (2) assess the gains in reliability of genomic predictions in Chinese Holstein from using a joint Chinese and Nordic reference dataset, compared with using the Chinese reference dataset alone.

## Methods

### Data

In this study, both the Chinese Holstein (CH) and Nordic Holstein (NH) cattle were genotyped with the Illumina BovineSNP50 BeadChip (Illumina, San Diego, CA). The single nucleotide polymorphism (SNP) data of each population were edited separately by deleting SNP with minor allele frequencies less than 0.01 or call rates less than 0.10, and excluding individuals with more than 10% missing marker genotypes. After editing, 41 838 SNP on 29 autosomes were retained in both populations. The genotyped CH cattle included 80 bulls born between 1993 and 2002 and 2091 cows born between 2001 and 2006, which were daughters of 13 of the genotyped bulls. The number of daughters per bull ranged from 63 to 358, with a mean of 135. The genotyped NH cattle included 5216 bulls born between 1974 and 2008. All animals of both populations were used in the linkage disequilibrium analysis. Deregressed proofs (DRP) were used as phenotypes for genomic prediction. DRP of CH bulls and cows were derived from the estimated breeding values (EBV) obtained from the Chinese genetic evaluations in April 2012 (Dairy Association of China), and DRP of NH bulls were derived from the EBV of Nordic genetic evaluations in November 2010 (Nordic Genetic Evaluation). Three traits (milk yield, fat yield and protein yield) were analyzed. In total, 4398 NH bulls and all CH animals had phenotypes for the three traits. 512 CH cows with possible incorrect sire information were discarded based on parentage verification with 255 SNP performed in a previous study [9] in which paternity was considered incorrect if five or more SNP were in conflict (i.e., a sire was homozygous for one allele but its daughter was homozygous for the other allele). Consequently, 1572 CH cows and 80 CH bulls with reliable pedigree information were used for genomic prediction.

### Measure of linkage disequilibrium (LD) and consistency of LD

Each chromosome was phased separately using Beagle [10] for each population, and all missing genotypes were simultaneously imputed by Beagle. Linkage disequilibrium between a pair of SNP was measured as $r_{\mathrm{LD}}^{2}$[11], and *r*_*LD*_ was calculated as follows:

$$r_{\mathrm{LD}}=\frac{f\left(\mathrm{AB}\right)-f\left(A\right)f\left(B\right)}{\sqrt{f\left(A\right)f\left(a\right)f\left(B\right)f\left(b\right)}},$$

where f(AB), f(A), f(a), f(B) and f(b) are observed frequencies of haplotype AB, alleles A, a, B and b, respectively. Maternal and paternal haplotypes were pooled to calculate LD. The consistency of LD in the two populations was measured by the correlation of *r*_*LD*_ of adjacent marker pairs on each autosome between the two populations [12].

### Prediction of genomic breeding values

A single-trait GBLUP model was used for genomic prediction based on the Chinese reference dataset, and a two-trait GBLUP was used for genomic prediction based on the joint reference dataset that included both the CH and NH cattle. In the latter, a single biological trait was regarded as a different trait in the two populations. The basic GBLUP model [13,14] was

y=1μ+Zg+e,

where **y** is the vector of phenotypes, *μ* is the population mean, **g** is the vector of breeding values, **e** is the vector of residuals, and **Z** is a design matrix allocating **g** to **y**. It was assumed that $g\sim N\left(0,G\sigma_{g}^{2}\right)$ and $e\sim N\left(0,D\sigma_{e}^{2}\right)$, where **G** is the genomic relationship matrix, $\sigma_{g}^{2}$ is the additive genetic variance, **D** is a diagonal matrix with weights of the residual variance [15], and $\sigma_{e}^{2}$ is the residual variance. The **G** matrix was constructed according to the method (method 1) described by VanRaden [14], i.e. **G** = **MM** '/∑2*p*_*i*_(1 - *p*_*i*_), where elements in column i of **M** are 0 - 2p_i_, 1 - 2p_i_ and 2 - 2p_i_ for genotypes A_1_A_1_, A_1_A_2_ and A_2_A_2_, respectively, and p_i_ is the allele frequency of A_2_ at locus i, calculated from the available marker data. Variances and covariances in the GBLUP models were estimated using the “average information” restricted maximum likelihood algorithm, and the GBLUP analyses were conducted using the DMU Package [16].

### Validation

Accuracy of genomic predictions using the CH and the joint reference datasets was assessed by two validation procedures. In the first procedure, the reference dataset comprised 13 genotyped CH bulls and their genotyped daughters and the test dataset consisted of 48 CH bulls without genotyped daughters. The remaining 19 CH bulls, which were highly related with the 13 reference bulls, were not used in the test dataset. In the second procedure, a five-fold cross-validation procedure was used for genomic prediction of CH cows. In each fold of cross-validation, two or three half-sib families of cows were used as the test dataset and the remaining cows and genotyped CH bulls were used as reference dataset. The numbers of animals in the reference and test datasets for the two validation procedures are shown in Table 1. Genomic EBV (GEBV) for the animals in a test dataset were predicted using both the CH and the joint reference datasets. The joint reference dataset included all 4398 Nordic bulls.

**Table 1:** The size of test and reference datasets used for validating genomic predictions of Chinese Holstein (CH) bulls and cows

**Categories**	**Test datasets**		Reference datasets^*^
CH bulls	48		13 bulls and 1572 cows
CH cows	set 1	337	80 bulls and 1235 cows
	set 2	309	80 bulls and 1263 cows
	set 3	323	80 bulls and 1249 cows
	set 4	307	80 bulls and 1265 cows
	set 5	296	80 bulls and 1276 cows

^* ^CH reference datasets; when using joint reference datasets for prediction 4398 Nordic Holstein bulls were included.

Reliabilities of GEBV for bulls and cows were measured as the squared correlation of GEBV and DRP divided by the average reliabilities of the DRP in the test dataset (Cor^2^_(GEBV,DRP)_/r^2^_DRP_) [15]. Because CH bulls were born between 1993 and 2002, genetic trend due to selection could inflate the correlation between GEBV and DRP. Therefore, in the validation of genomic predictions for CH bulls, genetic trends present in GEBV and DRP were corrected by regressing on birth year, and then the reliabilities were calculated by correlating the corrected GEBV and DRP. In the validation of genomic predictions for cows, reliabilities were calculated based on GEBV pooled over the five test datasets.

## Results and discussion

### Linkage disequilibrium and consistency of LD

**Figure 1:**
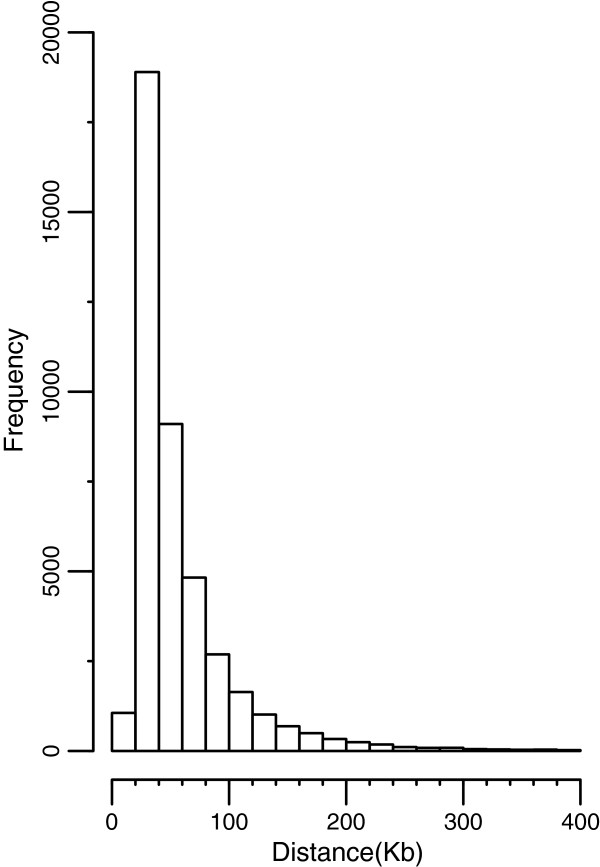
Frequency distribution of distances between adjacent SNP pairs.

Data on the 41 838 SNP distributed over the 29 bovine autosomes are summarized in Table 2. The average distance between adjacent SNP was 59.59 Kb, and the shortest and longest gaps were 3 bp (on BTA7) and 3820 Kb (on BTA10). As shown in Figure 1, most distances between adjacent markers were less than 100 Kb. However, 2.55% adjacent SNP had large distance gaps (larger than 200 Kb). This indicates that SNP used in the current study were not evenly distributed over the bovine chromosomes.

**Table 2:** **Distance and linkage disequilibrium (**$r_{\mathrm{LD}}^{2}$**) of adjacent SNP for each *Bos taurus *autosome (BTA)**

**BTA**	**Length (Mb)**	**Number of SNP**	**Mean distance (Kb)**	**Mean** $r_{\mathrm{LD}}^{2}$		**Consistency**^**1**^
				**Chinese Holstein**	**Nordic Holstein**	
1	160.89	2729	58.98	0.22	0.23	0.97
2	138.92	2221	62.58	0.22	0.22	0.98
3	125.41	2067	60.70	0.21	0.22	0.97
4	120.39	2031	59.30	0.20	0.21	0.97
5	124.59	1740	71.64	0.21	0.21	0.97
6	119.01	2099	56.73	0.22	0.23	0.97
7	112.37	1809	62.15	0.22	0.24	0.98
8	115.59	1940	59.61	0.21	0.22	0.98
9	104.64	1639	63.88	0.20	0.21	0.97
10	103.09	1752	58.87	0.22	0.22	0.97
11	106.97	1858	57.61	0.21	0.21	0.97
12	89.17	1338	66.70	0.19	0.20	0.97
13	83.84	1434	58.50	0.22	0.22	0.97
14	83.15	1409	59.06	0.24	0.25	0.98
15	83.81	1371	61.17	0.18	0.19	0.96
16	80.09	1283	62.47	0.22	0.23	0.98
17	74.89	1324	56.60	0.19	0.20	0.97
18	65.16	1101	59.24	0.19	0.19	0.96
19	62.83	1131	55.60	0.18	0.20	0.97
20	72.00	1314	54.84	0.20	0.21	0.97
21	68.46	1110	61.73	0.21	0.22	0.97
22	60.14	1047	57.50	0.19	0.19	0.97
23	51.73	895	57.86	0.17	0.18	0.97
24	62.77	1053	59.67	0.21	0.21	0.98
25	43.23	825	52.46	0.19	0.20	0.96
26	50.95	871	58.57	0.19	0.20	0.97
27	45.33	801	56.67	0.16	0.16	0.96
28	46.06	783	58.90	0.16	0.17	0.95
29	50.53	863	58.62	0.17	0.18	0.97
Mean	2506.00^2^	41 838^2^	59.59	0.20	0.21	0.97

^1^The correlation of *r*_*LD*_ of adjacent SNP pairs between two populations; ^2^Sum over 29 autosomes.

**Figure 2:**
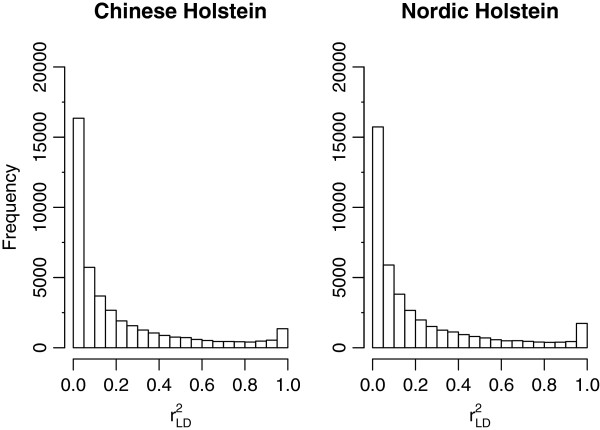
**Frequency distribution of **$r_{\mathrm{LD}}^{2}$**for adjacent SNP pairs in Chinese Holstein and Nordic Holstein populations.**

Figure 2 presents the distribution of $r_{\mathrm{LD}}^{2}$ of adjacent SNP pairs in the CH and NH populations. The LD between adjacent SNP pairs was generally small. The proportion of adjacent SNP pairs which had an $r_{\mathrm{LD}}^{2}$ < 0.01 was 15.2% for CH and 17.6% for NH; the proportion with an $r_{\mathrm{LD}}^{2}$ < 0.1 was 52.81% for CH and 51.72% for NH. These results indicate that the SNP markers did not effectively capture all the QTL affecting a trait, since most adjacent SNP pairs were in weak LD. The mean $r_{\mathrm{LD}}^{2}$ of adjacent SNP pairs within a chromosome ranged from 0.16 to 0.24 in CH and from 0.16 to 0.25 in NH. The mean $r_{\mathrm{LD}}^{2}$ across all chromosomes was 0.20 in CH and 0.21 in NH. The consistency of LD between the CH and NH populations was high (Table 2). The correlation of *r*_*LD*_ of adjacent SNP ranged from 0.95 to 0.98 across all chromosomes, with a mean of 0.97 at an average marker distance of 60 Kb.

The mean $r_{\mathrm{LD}}^{2}$ in the CH and NH populations was similar to the degree of LD reported for the Holstein populations in Germany [17], in the Netherlands, Australia and New Zealand [12], and in North America [18]. The consistency of LD between the CH and NH populations agreed with the consistency of LD between the Dutch and Australian Holstein bulls reported in [12] and was in line with the development of the CH population. The first dairy cattle imported in China came from Europe in the 1870’s [19] and since then, Holstein cattle have continuously been imported from Europe, Japan and North America. The imported Holstein bulls were crossed with local yellow cattle, and the crossbred cows were continuously back-crossed with the imported Holstein bulls [20]. The resulting crossed black and white dairy cattle were officially named Chinese Holsteins in 1992. Currently, most of the Holstein bulls found in China were imported from worldwide in the form of embryos or live cattle. Besides, the NH population has also exchanged genetic material with the United States, the Netherlands, Germany and other countries. The genomic relationship matrix showed that some CH bulls could be full-sibs or half-sibs of the NH bulls. Based on these data, it can be inferred that the Chinese Holstein population is genetically close to European and North American Holstein cattle.

### Genomic prediction

Reliabilities of GEBV for the 48 CH test bulls that had no genotyped daughters and the 1572 CH cows in the test dataset with the five-fold cross-validation are presented in Table 3. When the CH cattle were used as the reference data, the reliabilities of GEBV were 0.16 for CH test bulls and 0.14 for CH cows, averaged over the three traits. When using the joint reference dataset, the average reliabilities increased to 0.45 for CH bulls and to 0.21 for CH cows. Based on data from the Dairy Association of China, the reliability of a conventional pedigree index (calculated as 0.5*sire EBV + 0.25*maternal grandsire EBV) in the CH population was 0.12 for milk production traits. Thus, the reliabilities of genomic predictions based on the CH reference population were of similar magnitude as the reliabilities of a pedigree index, and the genomic predictions based on the joint reference population gave much higher reliabilities than those based on a pedigree index.

**Table 3:** Reliabilities of GEBV of Chinese Holstein (CH) bulls and cows in the test populations when using the CH or the joint reference population

**Categories**	**Traits**	**Reliabilities of prediction**		
		**Using CH reference**	**Using joint reference**	**Increase**
CH bulls	Milk yield	0.22	0.51	0.29
	Fat yield	0.15	0.47	0.32
	Protein yield	0.11	0.36	0.25
	Average	0.16	0.45	0.29
CH cows	Milk yield	0.15	0.26	0.11
	Fat yield	0.12	0.17	0.05
	Protein yield	0.15	0.20	0.05
	Average	0.14	0.21	0.07

The low reliabilities of genomic predictions using the CH reference dataset alone suggested that the information in the CH reference dataset, which mainly contained cows, was insufficient. According to Goddard [2], the reliability of GEBV for different sizes of reference population and different heritabilities of traits can be predicted as:

$$E\left(r_{\mathrm{GEBV}}^{2}\right)=1-\frac{\lambda }{2N\sqrt{a}}\times \log\frac{1+a+2\sqrt{a}}{1+a-2\sqrt{a}},$$

where *N* is the number of individuals in the reference population and *a = 1 + 2λ/N*. According to Hayes et al. [21], *λ = M*_*e*_*k/h*^*2*^, *M*_*e*_ *= 2N*_*e*_*L*, and *k = 1/log(2Ne),* where *N*_*e*_ is the effective population size and *L* is the length of the genome in Morgans. Using the above formula, the reliability of GEBV based on the CH reference dataset was expected to be 0.175, assuming *L* = 30, *Ne* = 100, *N* = 1500 and *r*^*2*^_*DRP*_ = 0.50. The reliabilities obtained from the validation procedures were consistent with these expected reliabilities. The results indicate that the size of the CH reference population needs to be increased in order to increase the reliability of genomic predictions.

Dairy cattle reference populations usually comprise progeny-tested bulls to maximize the information from each genotyped individual. In some countries or in some cattle populations where the number of progeny-tested bulls is small, one solution is to include cows in the reference population. In order to evaluate the value of adding cows to the reference dataset, an additional analysis was performed using a CH reference dataset from which 50% of the cows were deleted. The reliabilities of GEBV for the CH bulls using the reduced CH reference dataset decreased to 0.09, 0.03 and 0.05 for milk yield, fat yield and protein yield, respectively. This indirectly demonstrates that it is feasible to use cows as reference animals for genomic prediction, when the number of available progeny-tested bulls is not sufficient. A simulation study by Mc Hugh et al. [22] also suggested that genomic information from cows could greatly increase genetic gain and accuracy of male selection. To increase the size of the cow reference population at low cost, a good alternative would be to genotype cows using a low density chip like the Bovine LD (7 K) and then impute the genotypes for the 54 K panel.

The joint reference dataset greatly improved the reliability of genomic predictions for the CH cattle. The reliabilities of GEBV for CH bulls based on the joint reference dataset were close to those for NH bulls based on the Nordic reference data [23]. Several studies have reported that the reliability of genomic prediction can be increased by using a joint reference dataset that includes reference animals from other populations. The reliabilities of GEBV increased by 10% on average when four European Holstein populations were combined into a reference dataset, compared to when only one national population was used as the reference population [5]. Reliabilities of genomic prediction for Canadian Holstein bulls increased by 6% on average when about 3000 foreign bulls were included in the reference dataset [4], and by 7% when all North American sires were included [24]. Reliabilities were 2.6% higher for Holstein and 3.2% higher for Brown Swiss cattle when 3593 foreign Holstein and 732 foreign Brown Swiss animals were included in the reference dataset of the USA domestic prediction [6].

With the joint reference dataset, reliabilities of genomic predictions improved more for CH bulls than for CH cows i.e. by 2.3 fold for CH bulls and only by 1.7 fold for CH cows. This is due to the fact that CH bulls have a closer relationship with NH bulls than the CH cows do. Among the 48 CH test bulls, 14 bulls had a genomic relationship with one or more NH bull in the range from 0.45 to 0.56. However, no CH cow had this level of relationship with any NH bull. Moreover, among the 48 CH test bulls, 33 (68.75%) had a genomic relationship greater than 0.2 with at least one NH bull (with 15.5 bulls on average). Among the 1572 CH cows, only 459 (29.2%) had a genomic relationship greater than 0.2 with an NH bull (with 1.3 bulls on average). Many previous studies reported that the existence of a close relationship between test animals and reference animals increased the reliability of genomic predictions for the test animals [25-27].

To avoid overestimation of the reliability of GEBV, 19 CH bulls were excluded from the test dataset because they were highly related to 13 bulls in the reference population. In the five-fold cross-validation for the CH cows, two or three half-sib families were randomly assigned to a single-test dataset, instead of randomly choosing individuals. This was done to avoid overestimation of the reliability of GEBV when animals in the test dataset have a large group of half-sibs in the reference dataset. Moreover, genetic trend can increase the correlation between GEBV and DRP if the birth years of the animals in the test dataset cover a wide range. Therefore, in the validation for the CH bulls, the correlation between GEBV and DRP was calculated after correcting for genetic trend. When ignoring this correction, the validation reliabilities of genomic predictions for CH bulls using the joint reference dataset were unrealistically high at 0.69, 0.54 and 0.60 for milk yield, fat yield and protein yield, respectively.

In the current study, when using the joint reference dataset, genomic predictions were estimated using a two-trait model, in which the same biological trait was considered to be a different trait in the CH and NH populations. The reason for using a two-trait model, instead of a single-trait model, was that the DRP had different scales in the two populations due to the use of a standardization procedure in the NH population. The two-trait model also accounts for the presence of any genotype by environment interactions. When genotypes of bulls from three foreign countries were included in the USA domestic predictions, multi-trait methods were not more accurate than the single-trait model for Holstein cattle, but gave higher reliabilities (1.4% higher on average) for Brown Swiss cattle [6]. The authors suggested that this could be due to lower genetic correlations of traits between Brown Swiss populations. Using the two-trait GBLUP model, the estimated genetic correlations between the CH and NH populations were 0.85, 0.70 and 0.75 for milk yield, fat yield and protein yield respectively, which were much lower than the value of the consistency of LD of neighboring markers, which was 0.97 between the two populations. Assuming that the consistency of LD was appropriate to represent the genetic associations between different populations, its clear difference with the estimated genetic correlations suggests the existence of a large genotype by environment interaction between China and Nordic countries.

## Conclusions

The consistency of LD is very high between the CH and NH populations, indicating a high level of genetic similarity between the two populations. Genomic prediction for CH cattle can be greatly improved using a joint reference dataset that includes CH and NH cattle. In order to obtain satisfactory reliabilities of genomic predictions for CH cattle, it is necessary to increase the size of the CH reference population or to include foreign Holstein cattle in the reference population.

## Competing interests

The authors declare that they have no competing interests.

## Authors’ contributions

LZ performed the analysis and drafted the manuscript with help from GS. XD prepared Chinese Holstein data and helped in analyzing the data. GS, MSL, QZ and YW conceived and designed the study. MSL and GS helped in interpreting results. All the authors read, revised and approved the final manuscript.
